# Tumor-induced osteomalacia combined with increased bone resorption postoperatively: A case report

**DOI:** 10.1097/MD.0000000000034217

**Published:** 2023-07-07

**Authors:** Lingfeng Shi, Mengjia Tang, Shanshan Duan, Fang Deng, Yuping Zhang, Jing Yang, Jiongyu Hu

**Affiliations:** a Endocrinology Department, First Affiliated Hospital of Third Military Medical University (Army Medical University), Chongqing, China.

**Keywords:** bone pain, case report, fibroblast growth factor 23, muscle spasms, osteoporosis, tumor-induced osteomalacia

## Abstract

Rare tumor-induced osteomalacia (TIO) usually resulted in bone pain, fragility fractures and muscle weakness in clinical, which is caused by the reduced phosphate reabsorption, thus impaired mineralization of the bone matrix and free energy transfer. The specific problems in postsurgical patients are obscure although surgical removal of the tumor is the only definitive treatment. Here, we documented a female TIO patient who suffered more severe bone pain and muscle spasms post-operation. Further, we presented and discussed our explanation for the unexpected symptoms.

**Patient concerns:**

The main symptoms were whole-body pain and muscle weakness. The patient also presented with osteoporosis and multiple fractures.

**Diagnosis:**

Elevated serum fibroblast growth factor 23 (FGF23) level and hypophosphatemia indicated the diagnosis of TIO. Positron emission tomography (PET)/computed tomography (CT) with 68 Ga-DOTATATE located the tumor in the dorsolateral part of the left foot. Histopathological examinations confirmed the diagnosis.

**Interventions:**

The tumor was surgically removed immediately after the diagnosis of TIO and localization of the tumor. Postoperatively, calcium carbonate supplement treatment was continued.

**Outcomes:**

Two days after surgery, the serum FGF23 level was decreased to the normal range. Five days after surgery, N-terminal propeptide of type I procollagen and β-CrossLaps (β-CTx) had a remarkable increase. A month after surgery, the patient N-terminal propeptide of type I procollagen and β-CTx levels were decreased obviously, and serum FGF23, phosphate and 24h urinary phosphate were in the normal range.

**Lessons:**

We report a female patient who presented with osteoporosis and fractures. She was found with an elevation of FGF23 and diagnosis with TIO after PET/CT scanning. After surgically removing the tumor, the patient experienced more severe bone pain and muscle spasms. Active bone remodeling might be the reason for the symptoms. Further study will reveal the specific mechanism for this abnormal bone metabolism.

## 1. Introduction

Tumor-induced osteomalacia (TIO) is a rare paraneoplastic syndrome characterized by autonomous excessive secretion of fibroblast growth factor 23 (FGF23).^[1]^ The overproduced FGF23 acts on the proximal renal tubule to decrease the level of sodium phosphate cotransporters NaPi-2a and NaPi-2c, resulting in reduced phosphate reabsorption. Furthermore, 25-hydroxyvitamin D3 1-alpha-hydroxylase in the proximal tubules is also can be suppressed by FGF23, which could cause the reduction of 1,25(OH)_2_D and consequently reduce the intestinal absorption of phosphate and calcium.^[2,3]^

The main clinical features of TIO include bone pain, muscle weakness and fractures.^[4]^ With similar clinical symptoms and the age of onset to primary osteoporosis, as well as the extremely low incidence of the disease, the diagnosis of TIO is challenging.^[5–7]^ The previous investigations noted that the duration from the onset of symptoms to the diagnosis of it was a median of 2.9 years in China^[8]^ and 2.4 years in Japan.^[9]^ Here, we document a TIO patient who was considered as having primary osteoporosis for nearly 2 years and had an ineffective treatment. After the diagnosis of TIO and surgical removal of the tumor, the patient presented unexpectedly increased bone resorption, as well as more severe bone pain and muscle spasms. Although intensive care during the surgery was mentioned, the specific problems associated with bone metabolism disorders in postsurgical patients are poorly described in the literature.

## 2. Case presentation

The patient is a 43-year-old female who was suffering from 2 years of whole-body pain and muscle weakness. Her medical history included controlled type 2 diabetes (HbA1c 7.2%) for 2 years and well-controlled hypertension (SBP < 140 mm Hg or DBP < 90 mm Hg) for 6 years. She was on acarbose, sitagliptin, and amlodipine. She had no history of smoking, alcohol, or illicit drug use. Skeletal growth had been normal and her menstrual cycles were regular. She had not previously suffered from bone fractures or abnormal skeletal pains. She did not have a family history of skeletal, metabolic or hormonal disorders.

**Figure 1.**
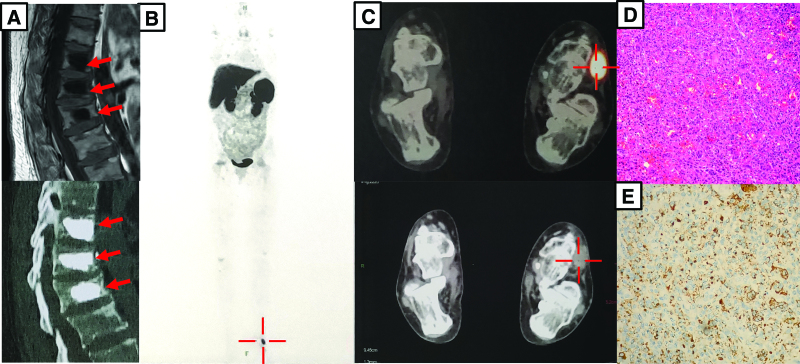
Radiological and pathological images of the patient. (A) Compression fractures and vertebroplasty of the spine in CT. The red arrows show the fractures and the bone cement. (B) The whole-body and feet (C) imaging of positron emission tomography (PET) computed tomography (CT) with 68 Ga-DOTATATE. The red cross shows the agent concentration on the left foot. (D) H&E staining and FGF23 immunochemical staining (E) of the tumor.

Two years prior to diagnosis, the patient visited a local hospital due to back pain and was diagnosed with primary osteoporosis. However, with 1-year calcium and vitamin D supplements treatment (calcium carbonate 600 mg and alfacalcidol 0.5 µg daily), the back pain was worse and followed by hip pain and muscle weakness. Thus, the patient went to the hospital again in 1 year later and found vertebral compression fractures in T11 to L1 vertebras. To ease the back pain, she experienced vertebroplasty with bone cement augmentation (Fig. 1A). Despite this, the back pain could not effectively treat and the patient gradually became unable to stand and move. Therefore, the patient visited the orthopedics outpatient clinic of our hospital 4 months before the correct diagnosis of TIO. The bone scanning indicated multiple rib fractures, bilateral sacral fractures, bilateral femoral neck fractures, and ilium fractures, with bone bruises in main joints such as the knee, elbows, wrists and intertarsal joints. Thought of the possibility of metabolic bone disease, the patient was referred to us by the orthopedic surgeon. At this point, the patient was bedridden and required continuous administration of pain drugs including various nonsteroidal anti-inflammatory drugs or oral tramadol.

Considering that the patient presented with severe osteoporosis, we measured the patient bone metabolism and calcium-phosphorus homeostasis at first. Then, parathyroid hormone (PTH) and creatinine in serum were tested due to hypophosphatemia. The results shown in Table 1, her serum PTH, creatinine, calcium and vitamin D were all in the normal range. Thus, after primarily excluding vitamin D deficiency, renal impairment, or hyperparathyroidism which could lead to hypophosphatemia in the patient, and further ruling out low-phosphate diet, hepatic diseases or intestinal disease which could cause decreased phosphorus uptake, we suspected the patient was suffering from acquired hypophosphatemic osteomalacia.

**Table 1:** Biochemical findings at diagnosis.

Parameter	Value	Reference range
Total calcium (mmol/L)	2.21	2.2–2.65
Inorganic phosphorus (mmol/L)	0.53	0.81–1.55
25-(OH)D (ng/mL)	23.16	˃20
1,25-(OH)2D (pmol/L)	27.40	25–200
β-CTx (ng/mL)	0.144	0.03–0.573
PINP (ng/mL)	46.24	15.13–58.59
PTH (pg/mL)	48.31	<70
BALP (µg/L)	29.71	0–14.3
N-MID Osteocalcin (ng/mL)	14.83	11–43
24 h urinary calcium (mmol/24 h)	1.85	0–6.25
24 h urinary phosphorus (mmol/24 h)	5.14	12.9–42
Creatinine (µmol/L)	35.3	45–84

1,25-(OH)_2_D = 1, 25-dihydroxyvitamin D, 25-(OH)D = 25 hydroxyvitamin D, BALP = Bone alkaline phosphatase, N-MID Osteocalcin = N-terminal mid-segment osteocalcin, PINP = N-terminal propeptide of type I procollagen, PTH = Parathyroid hormone, β-CTx = β-CrossLaps.

TIO is the most prevalent form of acquired hypophosphatemic osteomalacia. To test this, we further measured the serum FGF23 level and found it remarkably elevated (Table 2). Next, Positron emission tomography (PET) computed tomography (CT) with 68 Ga-DOTATATE (68 Ga-DOTA) was performed to image neuroendocrine tumors in the patient. Luckily, A 16 × 14 mm nodular shadow with imaging agent concentration was found in the dorsolateral part of the left foot (Fig. 1B and C), and further pathological examination of the puncture showed giant cell aggregation (Fig. 1D). Therefore, the diagnosis of TIO was made.

**Table 2:** Serum FGF23, phosphate, 24 h urinary phosphate, β-CTx, Osteocalcin*, PINP* and BALP concentrations during the hospitalization and 1 month follow-up.

Parameter (reference range)	Preoperative	The second postoperative d	The fifth postoperative d	One mo postoperatively
FGF23 (23.3–95.4 pg/mL)	266.3	15.7	NA	13.1
Total calcium (2.2–2.65 mmol/L)	2.21	2.25	2.35	2.38
24 h urinary calcium (0–6.25 mmol/24 h)	1.85	1.53	NA	2.14
Serum phosphate (0.81–1.55 mmol/L)	0.53	1.07	1.21	1.62
24 h urinary phosphate (12.9–42 mmol/24 h)	5.14	2.26	NA	37.06
β-CTx (0.03–0.573 ng/mL)	0.144	NA	3.610	0.714
N-MID Osteocalcin (11–43 ng/mL)	14.83	NA	28.89	17.37
*PINP* (15.13–58.59 ng/mL)	46.24	NA	117.10	52.61
BALP (0–14.3 µg/L)	29.71	NA	23.34	14.74

BALP = bone alkaline phosphatase, FGF23 = fibroblast growth factor 23, N-MID Osteocalcin = N-terminal mid-segment osteocalcin, PINP = N-terminal propeptide of type I procollagen, β-CTx = β-CrossLaps.

After the diagnosis was confirmed, the surgical removal of the tumor was operated. Histopathological examination of formalin-fixed and paraffin-embedded slides demonstrated a 16 × 14 mm phosphaturic mesenchymal tumor with multinucleated, osteoclast-like cells, with minimum tumor-free resection margins of 0.5 mm. Immunohistochemical staining showed a positive FGF23 staining (Fig. 1E), which is consistence with phosphaturic mesenchymal tumors and the diagnosis of TIO.

Postoperatively, oral neutral phosphorus supplement treatment (2g elemental phosphorus daily) was warranted to correct for severe hypophosphatemia. Administration of calcium carbonate 600 mg and alfacalcidol 0.5 μg daily was continued. Two days after surgery, the serum FGF23 concentration of the patient was decreased to 15.7 pg/mL (reference range 23.3–95.4 pg/mL). The serum phosphate and 24 h urinary phosphate were 1.07 mmol/L and 2.62 mmol/24 h, respectively (Table 2). Those demonstrated excellent postoperative results.

However, 5 days after surgery, the patient developed severe muscle cramps and spasms in all of her limbs, followed by persistent bone pain in her ribs, hipbones and long bones of the extremities. The serum calcium or phosphate concentrations of the patient were in the normal range, and the increased N-terminal propeptide of type I procollagen indicated a well bone formation after surgery. However, it is unexpected that the bone resorption marker β-CrossLaps (β-CTx) also had a remarkable increase compared to the pre-operation (Table 2). We used calcium gluconate intravenously to treat muscle spasms and found both the muscle spasms and bone pain could both alleviate by it. Thus, we increased the calcium carbonate to 1200mg per day. Ten days after surgery, the muscle cramps and bone pain were obviously alleviated and the patient decides to leave the hospital. A month after surgery, the patient N-terminal propeptide of type I procollagen and β-CTx levels were decreased obviously. Serum FGF23, phosphate and 24h urinary phosphate were in the normal range. The patient did not have muscle cramps or palpable bone pain and could do some exercise with assistance.

## 3. Discussion

Here, we report a 43-year-old female with TIO, who presented with back pain at the beginning and rapidly progressed to multiple fractures and muscle weakness. There was little benefit from calcium and vitamin D supplements treatment before the removal of the tumor, and neither got remission from vertebroplasty. After the patient was delivered to us, we found the patient had hypophosphatemia with normal serum calcium, PTH and creatinine level. Then, we measured the serum FGF23 level, and the elevated of it assisted us to diagnose TIO. Further, PET/CT with 68 Ga-DOTATATE helped us to locate the tumor. Interestingly, after the removal of the tumor in the dorsolateral part of the left foot, the patient emerged with severe bone pain and muscle spasms, which could be alleviated with the treatment of calcium.

TIO is a rare paraneoplastic syndrome, which could stimulate urine phosphorus excretion, leading to the gradual resorption of bone, and resulting in secondary osteoporosis, osteomalacia and fractures.^[10]^ A Japanese study documented that the average age of the TIO patients for the first visit to treating physicians was 52 ± 17 years,^[9]^ which is similar to the perimenopausal age. Therefore, TIO is easily be misdiagnosed as primary osteoporosis and missed. In our case, the patient was first considered as having primary osteoporosis and experienced ineffective calcium and vitamin D supplements treatment for a year. More importantly, the calcium and vitamin D treatment without the supplement of inorganic phosphorus possibly increased the risk of kidney stones and renal impairment.^[11]^

The autonomous secretion of FGF23 by tumor is the key modulator that causes urine phosphorus excretion. Thus, the elevation of FGF23 level in serum is a central role in the diagnosis of TIO.^[12,13]^ However, TIO is a rare disease with an approximate annual incidence of 0.04 per hundred thousand people.^[9]^ The low prevalence of TIO and the low awareness of it makes the measurement of FGF23 still not accessible in many areas. During the early treatment, our patient presented an unexpected acceleration in bone degradation, which led us to suspect the diagnosis of primary osteoporosis. Luckily, serum FGF23 could be measured in our hospital, and the elevated level of it helps us distinguish TIO from other secondary osteoporosis as Fanconi syndrome or vitamin D deficiency. Next, the tumor location is the second bottleneck in the diagnosis of TIO. Previous research indicated PET/CT with 68 Ga-DOTATATE as the first recommendation,^[14,15]^ which showed very high sensitivity (87.5%) to detect tumors regardless of tumor size.^[9]^ Nevertheless, we have received 3 adults presented as high FGF23 related hypophosphatemic osteomalacia with no mutation in the *dmp-1, enpp1, fgf23, phex*, and *slc34a3* genes in the past 2 years. This patient is the only one who successfully detected the tumor by 68 Ga-DOTATATE PET/CT.

Two days after the removal of the tumor, the patient serum FGF23 level was back to normal and hypophosphatemia was corrected. However, 5 days after the surgery, the patient appeared severe muscle spasms in all of her limbs. At this point, the patient was on calcium, vitamin D, and phosphate supplement therapy. Her serum calcium, phosphate, and albumin-corrected serum calcium were all in normal range. It seems that she was developing hypocalcemic muscle cramps except the serum calcium level did not decrease. Besides, she was also suffering a heavier bone pain in the whole body staring on the fifth postoperative day, and we found a significantly elevation of her serum β-CTx level. Surprisedly, the results indicates increased bone resorption after the removal of the tumor. To alleviate the spasms, we used calcium gluconate intravenously and it turned out to be effective on both spasms and bone pain. To explain this, we had a speculation. At the initial stage of TIO, the patient bone resorption was active due to hypophosphatemia. With the process of the disease, the persistence of bone resorption finally caused bone depletion and bone homeostasis sustained at a low background level. However, after the removal of the tumor, bone formation was active because of the over-reduced bone mass, fracture healing, calcium, and vitamin D supplementation therapy or the protective effect of estrogen. During the active bone remodeling process, the bone requires calcium eagerly. Thus, more serum calcium was used for bone formation and less calcium is available in the tissue fluid. Therefore, the patient was present a kind of hypocalcemic muscle cramps that can be alleviated by calcium supplements. Furthermore, bone resorption was also enhanced as a result of active bone remodeling, causing the elevation of β-CTx level and temporary bone pain. To treat the muscle cramps and promote bone formation, we double the dose of calcium carbonate daily to 1200 mg. As we expected, the symptoms were attenuated 3 days after adjusting and recovered well during the 1-month follow-up.

Conclusively, we report a TIO patient presented with osteoporosis with multiple fractures and appeared unexpected muscle cramps and bone pain post-operation. An explanation for the unexpected symptoms was also presented and discussed by us. Though we seem effectively treated the symptoms, the specific mechanism of it still unknown and worth to be further exploring.

## Author contributions

**Conceptualization:** Lingfeng Shi, Mengjia Tang, Yuping Zhang, Jiongyu Hu.

**Data curation:** Lingfeng Shi, Mengjia Tang, Jiongyu Hu.

**Formal analysis:** Lingfeng Shi, Mengjia Tang, Fang Deng, Jiongyu Hu.

**Funding acquisition:** Jiongyu Hu.

**Investigation:** Lingfeng Shi, Shanshan Duan, Yuping Zhang.

**Methodology:** Lingfeng Shi, Shanshan Duan, Fang Deng, Jing Yang.

**Project administration:** Lingfeng Shi.

**Resources:** Lingfeng Shi, Fang Deng.

**Software:** Lingfeng Shi, Jing Yang.

**Supervision:** Lingfeng Shi.

**Validation:** Lingfeng Shi.

**Visualization:** Lingfeng Shi.

**Writing – original draft:** Lingfeng Shi, Mengjia Tang, Jiongyu Hu.

**Writing – review & editing:** Lingfeng Shi.
